# Delayed contrast-enhanced cardiac MRI at an open 1.0T MR-system comparison of conventional segmented 3D gradient echo and phase-sensitive inversion recovery sequences: initial results

**DOI:** 10.1186/1532-429X-14-S1-P301

**Published:** 2012-02-01

**Authors:** Ortrud Kosiek, Frank Fischbach, Bernhard Schnackenburg, Alexander Schmeisser, Jan Smid, Skadi Wilhelmsen, Uta Wonneberger, Katharina A Strach

**Affiliations:** 1 Department of Radiology and Nuclear Medicine, University of Magdeburg, Magdeburg, Germany; 2 Clinical Science, Philips Healthcare, Hamburg, Germany; 3 Department of Cardiology, University of Magdeburg, Magdeburg, Germany

## Background

Delayed contrast enhanced (DE) cardiac MR imaging (cMRI) has become the gold standard for the in vivo detection and quantification of myocardial infarcts (MI). Up to now, cMRI had to be performed in a closed bore environment due to technical constraints of the existing open low-field MR scanners. The introduction of an open 1.0T MR-system with high gradient performance has paved the way for cardiac imaging, which may be beneficial in critically ill and claustrophobic patients as well as during stress examinations and MR-guided interventions.

The aim of this study was to evaluate image quality, scar/myocardium-contrast and assess feasibility of delayed contrast enhanced cMRI using a 1) breath hold (BH) 3 dimensional (3D) Inversion Recovery (IR), 2) BH phase-sensitive IR (PSIR), and 3) a respiratory triggered (RT) PSIR sequence at an open MR-system.

## Methods

41 patients (11 female, 30 male; age 57+/-15 years) with known or suspected MI underwent viability imaging 10 min after i.v. application of Gd-DTPA (0.2 mmol/kg BW) at an open MRI system (Panorama HFO 1.0 Tesla, Philips Healthcare, Best, Netherlands). DE cMRI with complete coverage of the left-ventricular myocardium in short-axis (SA) slices was acquired using three different sequences and approaches: 1) A conventional k-space segmented 3D T1-weighted inversion-recovery-prepared gradient-echo sequence (in-plane resolution 1.33x1.33 mm2, slice thickness 5 mm) was performed. The inversion delay time TI was iteratively adjusted to null the signal from normal myocardium. Additionally, a fast multislice single-shot 3D PSIR sequence was performed in 2) BH technique (in-plane resolution 1.51x1.71 mm2, slice thickness 10 mm, SENSE factor 2 and 3) respiratory triggered (in-plane resolution 1.8x1.8 mm2, slice thickness 10 mm).

Image quality of DE was rated on a 4-point grading scale. Contrast (C=SIscar/SImyo) was calculated and area of DE was assessed in % of LV mass.

## Results

There was no statistical significant difference in image quality using BH 3D IR, BH PSIR and RT PSIR (3.71+/-0.46; 3.27+/-0.61, 3.36+/-0.87, p<0.05), respectively. No infarcts were missed and agreement between the three approaches regarding infarct extent (BH 3D IR 5.47+/-9.08% vs. BH PSIR 5.26+/-8.89% vs. RT PSIR 5.59+/-8.85%, p< 0.05) was high. Contrast for 3D BH IR, BH PSIR and RT PSIR was 10.4+/-3.3 and 11.8+/-3.0, and 12.6+/-1.9, respectively.

## Conclusions

Delayed contrast enhanced cMRI is feasible at an open 1.0T MRI system in a clinical setting and offers excellent image quality. Respiratory triggered PSIR findings correlate well with breath hold 3D IR and PSIR sequences for the detection and extent of myocardial infarcts.

## Funding

None.

**Figure 1:**
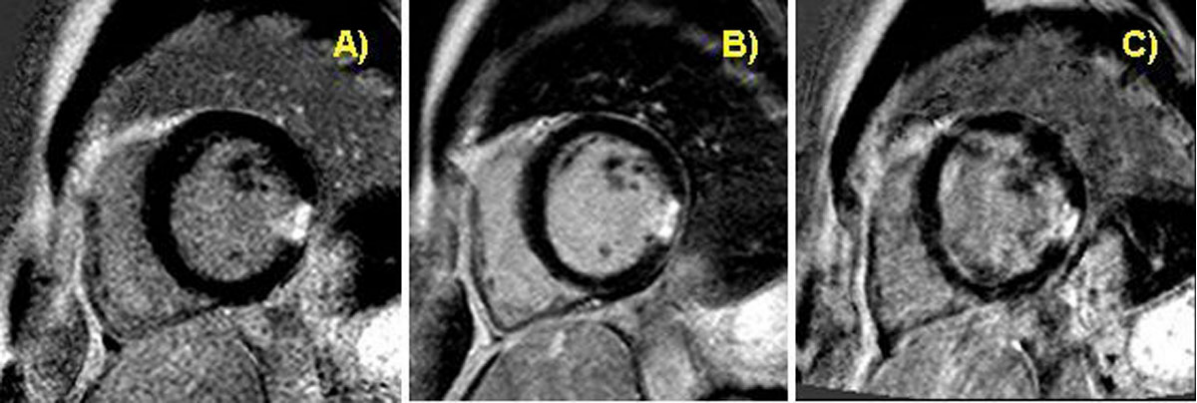
Small transmural infarct inferolateral in breath hold 3D inversion recovery (A), breath hold PSIR (B), and respiratory triggered PSIR (C) technique. Image quality was excellent using approaches (A) and (B). Due to an arrhythmic breathing pattern image quality was reduced however still diagnostic for approach (C).

